# Repair of the myometrial scar defect at repeat caesarean section: a modified surgical technique

**DOI:** 10.1186/s12884-021-04040-9

**Published:** 2021-08-17

**Authors:** Shahul Hameed Mohamed Siraj, Karuna Mary Lional, Kok Hian Tan, Ann Wright

**Affiliations:** 1 Department of Minimally Invasive Surgery, Division of Obstetrics and Gynaecology, KK Women’s and Children’s Hospital, Singapore, Singapore; 2 Department of Maternal Fetal Medicine, Division of Obstetrics and Gynaecology, KK Women’s and Children’s Hospital, 100 Bukit Timah Road, Singapore, 229899 Singapore; 3 Perinatal Audit and Epidemiology Unit, Division of Obstetrics and Gynaecology, KK Women’s and Children’s Hospital, Singapore, Singapore

**Keywords:** Residual myometrial thickness, Caesarean scar dehiscence, Myometrial repair

## Abstract

**Background:**

To investigate whether the existing surgical technique for uterine closure at repeat lower segment Caesarean section (LSCS) can be modified to achieve adequate residual myometrial thickness (RMT) to ensure scar integrity and reduce complications in future pregnancy.

**Methods:**

Women with a significant scar defect at repeat LSCS had the anterior uterine wall closed by a single experienced obstetrician with a technique focused on recognition, mobilisation and apposition of the retracted myometrial edges at the boundary of the defect. This was aimed at anatomical restoration of the lower segment. The RMT at the scar area was assessed by postnatal pelvic ultrasound scan at three months.

**Results:**

Thirty women with a history of at least one previous CS, incidentally found to have a large defect at operation underwent the technique with prior consent. A postnatal scan showed a mean residual myometrial thickness of 8.4 mm (SD ±1.3 mm; range 5.6–11.0 mm). The average operating time was 91 mins and the average blood loss 728 ml. Two women who underwent the repair have gone on to have a further uneventful CS.

**Conclusion:**

This modified technique resulted in scan evidence of an RMT indicative of uterine wall stability postnatally and offers the potential for reducing the risk of rupture and placenta accreta spectrum (PAS) in future pregnancy.

## Background

The myometrial niche and its subsequent development into a scar defect during pregnancy after caesarean section (CS) has been associated with various complications. The risk of uterine rupture increases with the size of defect, being higher with any scar thickness of ≤5 mm [1]. Abnormal placental implantation has also been linked to a large area of myometrial deficiency [2]. While niche repair has been performed for years for a variety of gynaecological indications and may be incorporated into the surgical management of scar pregnancy [3], apart from some highly specialised uterine conserving techniques used for placenta accreta spectrum (PAS) disorders, repair of the dehiscence at uncomplicated repeat CS has not been reported [4, 5].

The niche is an area of deficiency at the lower uterus which corresponds to the site of a previous operative delivery. It develops into an isthmocele as pregnancy progresses and may be classified as large or small depending on the wall thickness of the myometrial defect expressed as an absolute measurement or percentage. Niche incidence following prior CS has been reported as high as 84% using gel instillation sono-hysterography [4, 5]. No niche has been reported without previous CS although localized myometrial deficiency is a recognized complication of previous gynaecological surgery. The risk of niche enlargement increases with number of previous CS and possibly uterine retroflexion. The aetiology of the niche is not fully understood and uterine incision site, often related to labour characteristics, surgical closure technique (single or double layer, locking sutures, with or without inclusion of the decidua, amongst others) and patient factors have all been suggested as possible contributors [6]. Complications of previous Caesarean section on subsequent pregnancies have been comprehensively documented and include uterine rupture with potentially catastrophic consequences for both fetus and mother which may be as high as 5% with a large defect [7]. Antenatal ultrasound assessment of myometrial thickness appears to have limited use as a prognosticator for obstetric outcomes but is a marker for uterine wall stability after repair at caesarean section [8, 9].

Many studies have looked at factors favouring vaginal birth after caesarean section (VBAC) but when considering the anatomical changes at the previous scar, success of VBAC would be expected, at least in part, to depend not only on the size of the myometrial defect but more importantly, the strength of the overlying pelvic fascia and any attached muscle [8, 10]. Therefore, it is important during an elective or emergency repeat caesarean section to identify the retracted myometrial muscle and repair the defect to ensure integrity of the remaining scar.

### Altered anatomy of the lower uterus after caesarean section

During formation of the lower uterine segment (LUS) after previous CS, the myometrium at the niche may separate and retract resulting in a scar defect, the depth of which is related to the amount of muscle affected [6]. The inferior retracted muscle edges which become epithelialized can be difficult to recognize and might be missed during closure at subsequent operative delivery, usually performed in two layers. This leads to further deepening and widening of the defect with each CS until only fascia exists between the pregnancy and bladder forming the myometrial scar defect or isthmocele which is often referred to as “the thin lower segment” and repaired without including the underlying muscle layers. The myometrial defect can be identified on pelvic ultrasound as a niche especially in early pregnancy but MRI is useful in later pregnancy as it can help to identify the retracted muscle edges [2].

**Fig. 1:**
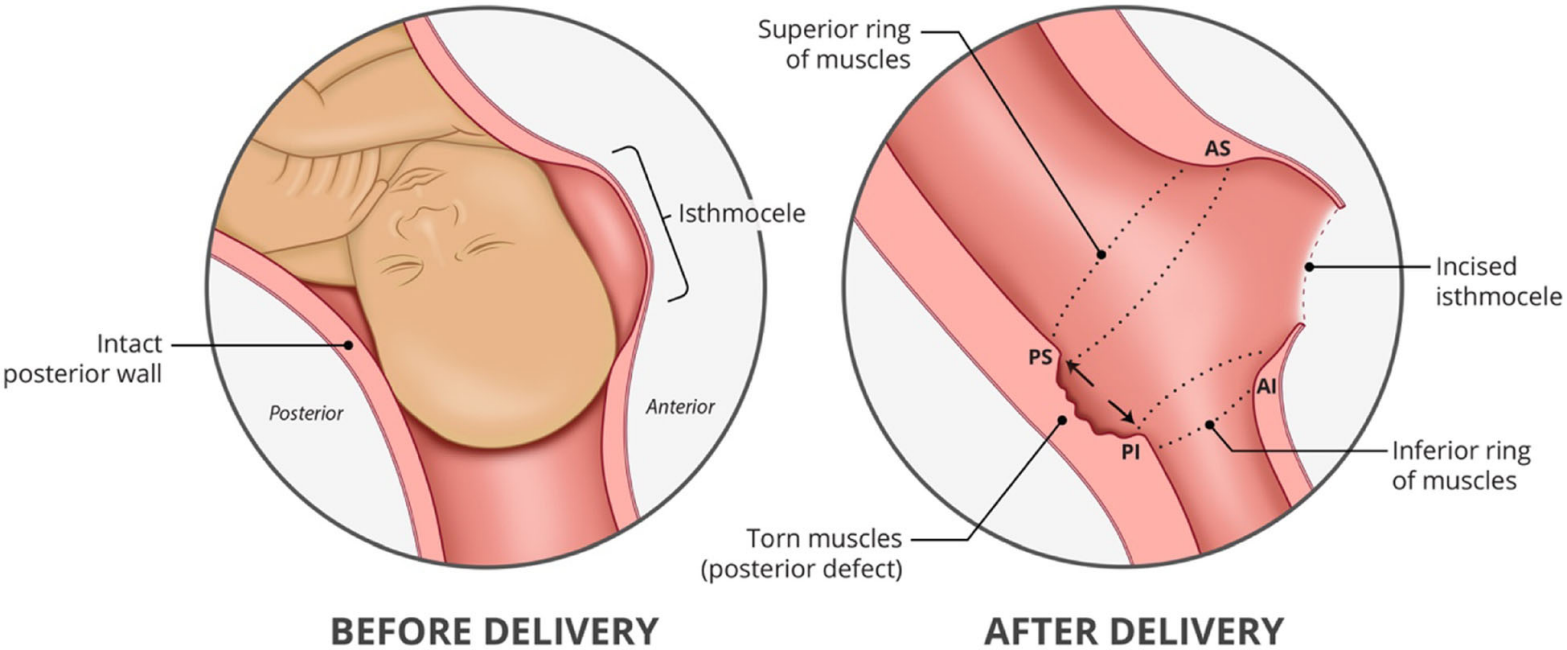
Recognition of the boundaries of the myometrial defect

We have observed that there is nearly always also thinning and tearing of the muscle layers in the posterior and lateral uterine walls which, we postulate, is due to the distribution of forces at the interface between the lower and upper uterine segments with advancing pregnancy (Fig. 1). We describe a technique which focuses on repair of the anterior uterine wall with recognition and correction of the myometrial defect resulting from a previous CS by mobilisation and apposition of the retracted muscle edges at the boundary of the defect allowing anatomical restoration and addresses any accompanying posterior wall damage and angle disruption.

## Methods

We wanted to evaluate the efficacy of a modified technique for the repair of the myometrial defect at repeat caesarean section. The study was conducted, as a pilot, in a tertiary obstetric referral hospital in Singapore which has around 12,000 deliveries per year and a caesarean section rate of 30–32%, between June and September 2019. All women having a repeat caesarean section done by a single senior obstetrician were counselled and written consent taken that if found to have a large scar dehiscence/ myometrial defect at surgery it would be formally repaired. Thirty women were identified at caesarean delivery to have a large dehiscence and underwent the repair with a postnatal scan organised at three months to measure RMT.

### Modified technique

The abdomen was opened through the previous scar. The visceral peritoneum at the utero vesical junction, was divided to reveal the scar defect below and the bladder was dissected free from the lower uterus. The uterus was opened transversely through the lower third of the scar defect by a horizontal incision which was gently extended laterally using the index fingers or scissors and the baby was delivered in the usual way. The uterus was then repaired in the following steps:

#### Step 1: Recognition of the retracted myometrium around the boundary of the myometrial defect

- Lower (inferior) ring of muscle (Refer Fig. 1)

The retracted anterior muscle edge at the inferior boundary of the defect was first sought. The bulk of this retracted lower myometrial muscle can be identified by running a finger up from the internal os superiorly to where the tissue first thins. This muscle band is grasped with Green Armytage clamps along its length. In cases of more than one previous CS where there may be more than one defect with several bands of muscle within the fascia, the lowermost band of muscle is followed laterally towards the angles and onto where it meets the attenuated muscle of the posterior uterine wall in the form of a ring. This allows all the retracted myometrium to be included in the repair.

b. Upper (superior) ring of muscle

The muscle at the upper anterior edge of the defect was identified and held with Green Armytage clamps and followed round posteriorly.

c. Identification of the posterior defect

Any additional posterior myometrial defect is then identified between the two muscle rings in the posterior uterine wall.

#### Step 2: Re-attachment of the myometrial defect (superior and inferior retracted muscle ring) and closure of the anterior uterus

After freshening, both the superior and inferior rings of muscle they were re-attached in the following order using a number polyglactin 910 1–0 suture on a round bodied needle: -

**Fig. 2:**
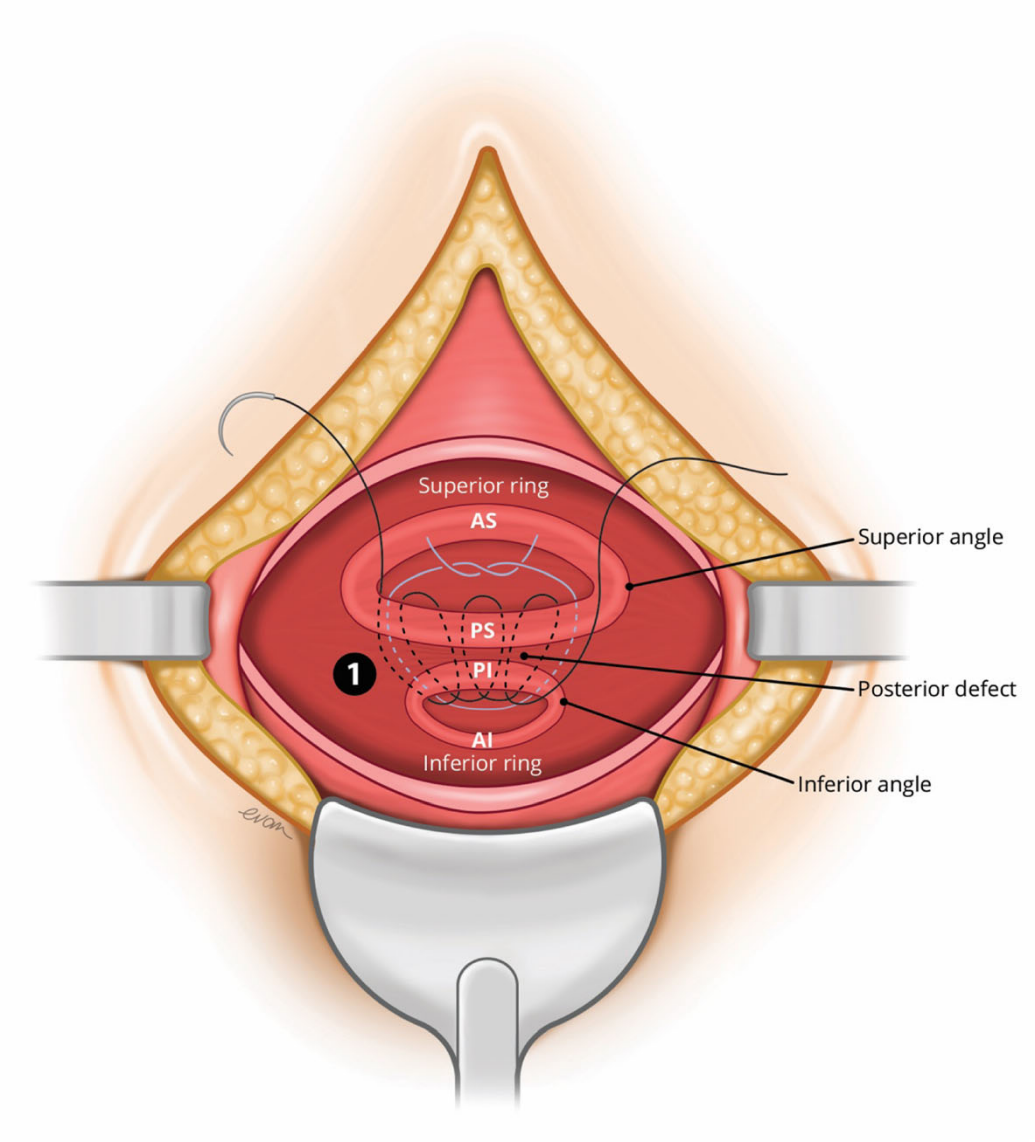
Repair of the posterior wall defect

- Repair of the posterior wall defect (Refer Fig. 2)

The torn posterior muscle edges were held between the two rings with clamps and placing a large horizontal mattress suture loosely to hold the muscle edges together before closing the gap between the edges with a continuous suture.

b. Reconstitution of the lateral angles (right and left) of the myometrial defect

**Fig. 3:**
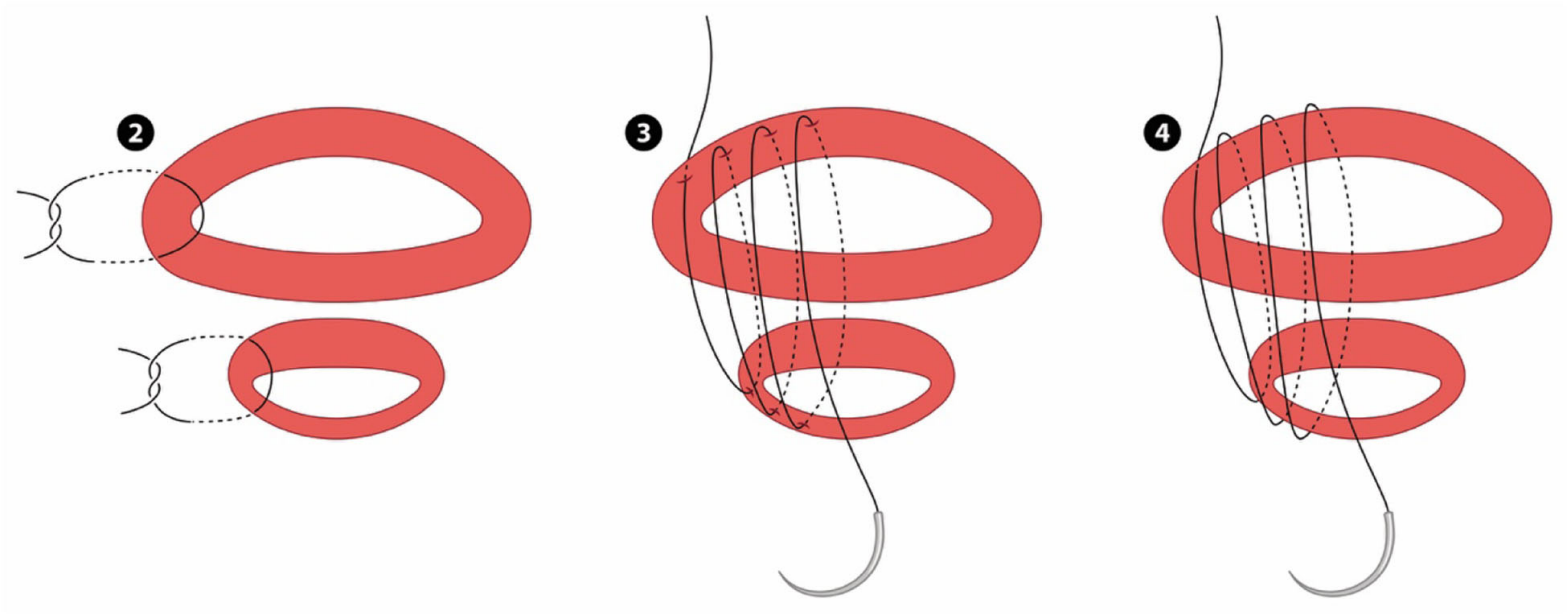
Repair of the angles and anterior defect

Both torn lateral angles were reconstructed by identification and re-attachment to their ipsilateral origin. This reduced the tension before bringing them together bilaterally using a figure of eight suture.

c. Closure of the antero-inferior and antero-superior boundary of the myometrial defect continuous suture (Refer Fig. 3)

**Fig. 4:**
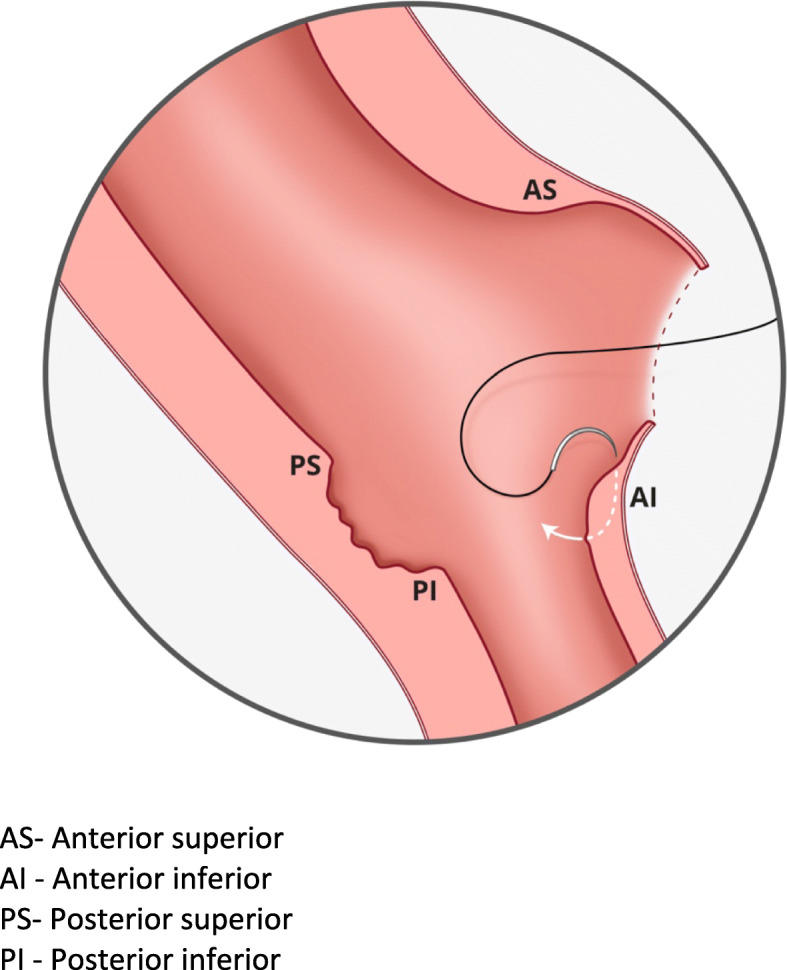
Needle placement for myometrial repair. AS- Anterior superior. AI - Anterior inferior. PS- Posterior superior. PI - Posterior inferior

This step could be likened to bringing together two ends of a tube (with a thin posterior wall which has already been repaired). The initial layer needs to pick up the muscle at the base of the myometrial defect by inserting the needle perpendicular to the bulk of the muscle before bringing it through to the cavity and subsequently through the superior muscle from inside out. (Refer Fig. 4) Closing this layer by suturing from both angles and tying in the middle reduces the inadvertent risk of closing off the lower cavity.

d. Completing the closure of the anterior uterine wall

**Fig. 5:**
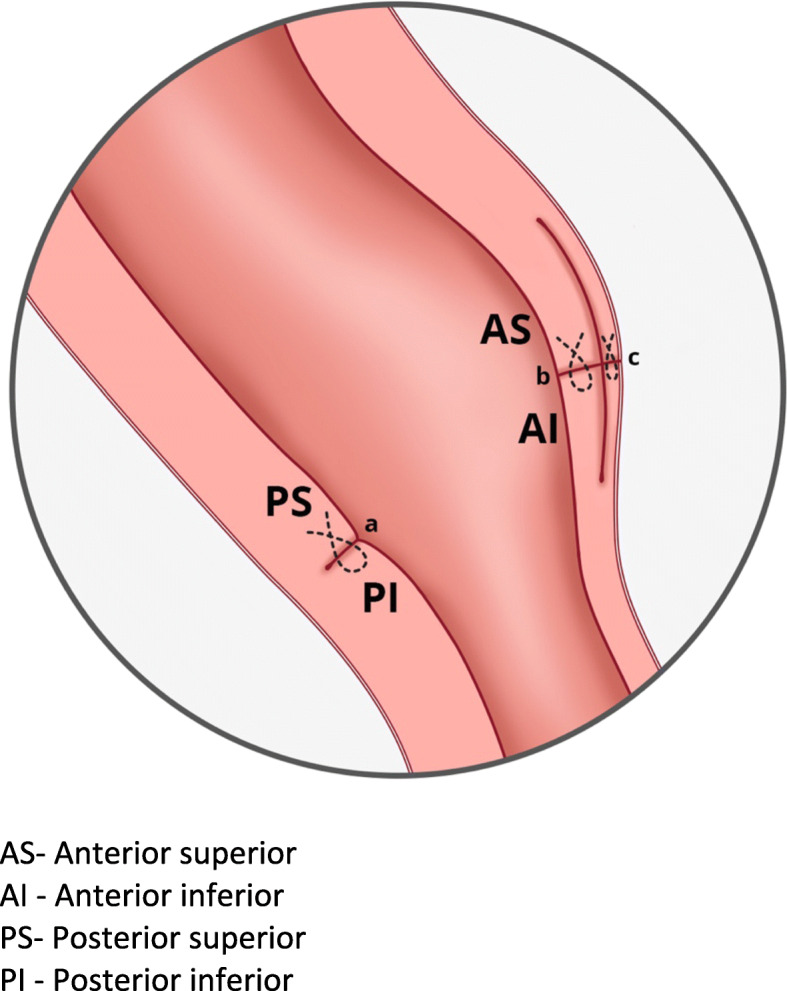
The appearance of anterior and posterior wall defect after repair. AS- Anterior superior. AI - Anterior inferior. PS- Posterior superior. PI - Posterior inferior. **a** continuous suture for posterior myometrial defect. **b** continuous suture for inner layer of anterior myometrial defect. **c** continuous suture for outer layer of anterior myometrial defect

The remaining muscles were then closed in two or three layers depending on the bulk of muscles suturing from the angles by approximation of the upper and lower myometrial edges with the aim of building up and equalizing the depth of the superior and inferior aspects of the anterior uterine wall musculature and incorporating the fascia. Refer to Fig. 5 which illustrates the final appearance of the anterior and posterior wall defects after repair.

Following the closure, haemostasis was checked, the peritoneal cavity cleaned and the abdominal wall closed in layers in the usual way with polyglactin 910 1–0 sutures to the rectal fascia, subcutaneous tissue and a subcuticular suture such as monocryl. Use of a drain and antibiotics were optional depending on the clinical situation. Post-operative care was the same as for all repeat Caesarean section.

This differs from the standard technique in which the uterus is usually opened transversely and, following delivery, closed in two layers to secure haemostasis with no particular attention to identification of the previously separated muscles as it requires the steps of formal identification and mobilization of the retracted lower segment myometrium. Mobilisation facilitates the recognition of the extent of the retraction in the anterior lower segment and allows recognition of the posterior wall shearing and defect both of which ensure optimal anterior and posterior approximation and closure of the lower segment myometrium.

## Results

A total of 30 women, with a mean age of 31.8 years (SD ± 4.8 years) and mean BMI of 27.2 kg/m^2^ (SD ± 4.8 kg/m^2^) consented to undergo the technique if warranted. Of the 30 women, 23 had one previous CS; 6 had two; and one had three previous CS. Most of them (25(83.3%)) had the CS at term for singleton pregnancies. Five were preterm (including twins at 36 weeks and an IUD at 28 weeks). The average operating time was 91mins (SD ±20 min) and the average estimated blood loss was 728 ml (SD ±379 ml). A postnatal pelvic ultrasound at three months showed no evidence of a niche in any case and an average residual scar thickness of 8.4 mm (SD ±1.3 mm; range 5.6–11.0 mm).

## Discussion

Although myometrial defect repair has been used for gynaecological indications and has been suggested pre-pregnancy to improve outcomes, our technique provides an opportunity to repair the defect at the time of repeat LSCS. This has potential benefits, both for future pregnancy with respect to scar rupture and possibly prevention of PAS development. It may also help in reducing the likelihood of scar pregnancy and gynaecological symptoms which are increasingly being attributed to the presence of a scar defect.

It is important to raise awareness that a ‘thin lower segment’ in repeat CS is actually a myometrial defect with overlying fascia, which should be reconstructed by identifying and approximating the retracted muscle edges at the time of CS, regardless of indication. Differentiating this uterine window with placenta just below the serosa, from a placenta percreta, on antenatal scan and intraoperatively is also key to avoiding unnecessary complex surgical procedures [11].

The underlying principle must be understood and incorporated into obstetric surgical training. Because it is performed within the uterine serosa the technique has a low risk of visceral injury. It can also be used in conjunction with other haemostatic measures such as Bakri balloon, internal iliac occlusion or application of a paracervical tourniquet to reduce blood loss. It includes preservation of the overlying fascia as well as attention to and repair of the posterior uterine muscle, if torn, to avoid this becoming an area of weakness in future pregnancies.

## Conclusion

This technique aimed at repairing the scar defect at the time of repeat Caesarean section resulted in a residual anterior myometrial wall thickness previously associated with good delivery outcomes with the potential to reduce future obstetric and gynaecological morbidity.. Awareness and recognition of the condition is important but skill in the technique is easily acquired through practice and we advocate it should be incorporated into basic obstetric surgical training.

## Data Availability

Data supporting the conclusions of the study is available with Dr. Shahul Hameed Mohamed SIRAJ, Department of Minimally Invasive Surgery, Division of Obstetrics and Gynecology, KK Women’s and Children’s Hospital, Singapore.

## Abbreviations

LSCS: Lower segment caesarean section
RMT: Retro myometrial thickness
PAS: Placenta accreta spectrum
VBAC: Vaginal birth after caesarean section
LUS: Lower uterine segment
AS: Anterior superior
AI: Anterior inferior
PS: Posterior superior
PI: Posterior inferior
